# Two-Dimensional VO_2_ Mesoporous Microarrays for High-Performance Supercapacitor

**DOI:** 10.1186/s11671-018-2557-7

**Published:** 2018-05-08

**Authors:** Yuqi Fan, Delong Ouyang, Bao-Wen Li, Feng Dang, Zongming Ren

**Affiliations:** 1 College of Geography and Environment, Shandong Normal University, 88 East Wenhua Road, Jinan, 250014 People’s Republic of China; 2 Institute of Environment and Ecology, Shandong Normal University, 88 East Wenhua Road, Jinan, 250014 People’s Republic of China; 3 School of Materials Science and Engineering, Wuhan University of Technology, 122 Luoshi Road, Wuhan, 430070 People’s Republic of China; 4 Key Laboratory for Liquid-Solid Structural Evolution and Processing of Materials, Shandong University, 17923 Jingshi Road, Jinan, 250061 People’s Republic of China

**Keywords:** Two-dimensional VO_2_, Mesoporous structure, Supercapacitor

## Abstract

Two-dimensional (2D) mesoporous VO_2_ microarrays have been prepared using an organic–inorganic liquid interface. The units of microarrays consist of needle-like VO_2_ particles with a mesoporous structure, in which crack-like pores with a pore size of about 2 nm and depth of 20–100 nm are distributed on the particle surface. The liquid interface acts as a template for the formation of the 2D microarrays, as identified from the kinetic observation. Due to the mesoporous structure of the units and high conductivity of the microarray, such 2D VO_2_ microarrays exhibit a high specific capacitance of 265 F/g at 1 A/g and excellent rate capability (182 F/g at 10 A/g) and cycling stability, suggesting the effect of unique microstructure for improving the electrochemical performance.

**Electronic supplementary material:**

The online version of this article (10.1186/s11671-018-2557-7) contains supplementary material, which is available to authorized users.

## Background

Supercapacitors are rechargeable electrochemical energy storage devices, which have emerged with great potential to provide one-order higher energy density and a much longer cycling life than batteries through the fast surface charge storage processes [1–3]. Supercapacitors can be divided into two types: mesoporous carbon-based electrical double-layer capacitors (EDL) and reversible Faradaic reaction (redox reaction)-based pseudocapacitors of metal oxides and/or conducting polymer [4]. Pseudocapacitance, which shows at least one-order higher capacitance than the EDL effect, has drawn increasing attention for the development of pseudocapacitors with similar energy density as that of batteries [5, 6]. However, pseudocapacitors often suffer from a low power performance and cycle life because Faradaic redox reactions are often limited by low surface area and low electrical conductivity [7].

Transition metal oxides (TMOs), such as RuO_2_ [8, 9], MnO_2_ [10, 11], Fe_2_O_3_ [12, 13], NiO [14, 15], SnO_2_ [16, 17], have been extensively investigated as electrode materials for supercapacitors. Among them, vanadium oxides (for example, V_2_O_5_, VO_2_, and V_6_O_13_) has been investigated as electrode materials for supercapacitors and Li/Na ion batteries because of their high specific capacity, variable oxidation states, low cost, and abundant storage [18–31]. VO_2_ has potentials to obtain high performance due to its higher electronic conductivity arising from a mixed-valence of V^3+/5+^ and good structural stability. Up to now, VO_2_/rGO [28, 29, 32], VO_2_/CNTs [30], and hydrogen-treated nanoporous VO_2_ have been reported with excellent pseudocapacitance properties [33]. Supercapacitors consisting of VO_2_/GO nanobelts possessed a capacitane value of 426 F/g at 1 A/g in the potential range of − 0.6 to 0.6 V [29]. VO_2_ nanoflake arrays deposited on a carbon matrix showed capacitance values of 485 F/g at 2 A/g [34]. VO_2_/CNT nanocomposites synthesized by atomic layer deposition exhibited a capacitance up to 1550 F/g [30]. In pure VO_2_ nanocrystals, insufficient control of its microstructure at nanoscale typically existed and thus leaded to unsatisfied capacitance and cycle performance. VO_2_ nanosheet-based electrode materials obtained a capacitance of 150 F/g at 1 A/g [34]. Pure nanoporous VO_2_ electrodes only exhibited capacitance values of 76 F/g at 1 A/g [33]. Microarray of VO_2_ nanowires obtained a capacitance value of 180 F/g at 1 A/g with good cycle performance [35]. These results suggest that VO_2_ with good electrical conductivity and designed porous structure are critical for achieving high performance.

We previously developed the toluene–water system for synthesis of nanocrystals. The nucleation of metal oxide nanocrystals occurred in the aqueous phase, and then, the nanocrystals were drawn into the organic phase through the adsorption of surfactants on the liquid interface under hydrothermal conditions. The morphology evolution of nanocrystals occurred in organic phase. Highly dispersed nanocrystals with narrow size distribution and uniform morphology, such as CeO_2_, Fe_3_O_4_, and Mn_*x*_O_*y*_, have been synthesized [36–39]. Although VO_2_ nanoparticles and thin films have been prepared through the hydrothermal method, the rational design of their crystallinity and microstructure are difficult to achieve [40–42].

In this work, a liquid interface-derived method was developed to fabricate the 2D microarrays of VO_2_. The 2D microarrays have a millimetric size with a thickness of about 1 μm and two different surfaces formed in organic–aqueous interface. The block unit of the 2D microarrays is the VO_2_ needle-like particles with a uniform mesoporous structure, in which the pore size is about 2 nm. Such unique architecture provides a short diffusion route for electrolyte ion and numerous channels for the access of electrolyte. Furthermore, low resistance is realized in the VO_2_ microarrays. Based on this unique structure, the 2D mesoporous microarrays exhibit excellent capacitance performance with high specific capacitance, good rate, and long life cycle stability.

## Methods

### Materials

V_2_O_5_, H_2_O_2_ (30%), toluene, oleic acid, and *tert*-butylamine were purchased from Sigma Aldrich. These chemicals were used as received without further purification. Deionized (DI) water through a Millipore system (Milli-Q) was used in all experiments.

### Preparation of 2D VO_2_ Microarrays

In a typical synthesis process, 7.5 ml H_2_O_2_ (30%) is added into 150 ml deionized water, and then, 0.534 g V_2_O_5_ is added into the solution; the suspension was stirred at room temperature until a dark golden yellow solution was obtained and used as the aqueous phase in this process. A mixed solution of 30 ml toluene, 12 ml oleic acid, and 1.5 ml *tert*-butylamine was used as the organic phase. The aqueous and organic solutions were poured into a 200-ml autoclave and heated a 200 °C for 48 h. The 2D VO_2_ microarrays were grown on the organic–aqueous interface and deposited in the aqueous phase. Under centrifugation, the synthesis and the 2D VO_2_ microarrays were collected from the aqueous phase. Finally, the as-collected 2D VO_2_ microarrays were dried at 200 °C for 2 h in vacuum.

### Material Characterization

The XRD patterns of the resulting products were collected by X-ray diffractometer (XRD, D5005HR) with CuKα radiation under a voltage of 40 kV and a current of 40 mA. The sample morphology was investigated by a transmission electron microscopy (TEM, JEM-2100F). The microscopic features of the samples were collected by field-emission scanning microscope (FESEM, SU-70) equipped with an X-ray energy-dispersive spectrometer (EDS). The surface composition was investigated by X-ray photoelectron spectra (XPS, ESCALAB 250). The Brunauer–Emmett–Teller (BET) surface area and porosity were determined by nitrogen adsorption-desorption isotherm measurements using a Micrometritics ASAP 2020 analyzer at 77 K.

### Electrochemical Characterization

The electrochemical characteristics were examined by an electrochemical analyzer system (CHI660D Shanghai Chenhua Apparatus, China) in a three-compartment cell. The working electrodes were comprised of 80 wt% of active material, 10 wt% of acetylene black (AB), and 10 wt% of polyvinylidene difluoride (PVDF). *N*-methyl-2-pyrrolidone (NMP) was used as a solvent. The mixed slurries were coated onto Ni foils and then heated at 80 °C overnight to remove the organic solvent. The electrolyte was 1 mol l^−1^ Na_2_SO_4_ solution. Cyclic voltammetry (CV) curves were recorded using an electrochemical workstation PARSTAT 2273 with different scan rates. The electrochemical impedance measurements were carried out at 10 mV ac oscillation amplitude over the frequency range of 10 to 0.01 kHz. The electrical conductivity was measured at room temperature by a ST-2258A digital four-point probe test system. Prior to the measurement, sample powders were compressed into a wafer with a thickness of 0.2 mm and a diameter of 13 mm by an oil pressure machine under a pressure of 30 MPa.

## Results and Discussion

**Scheme 1:**
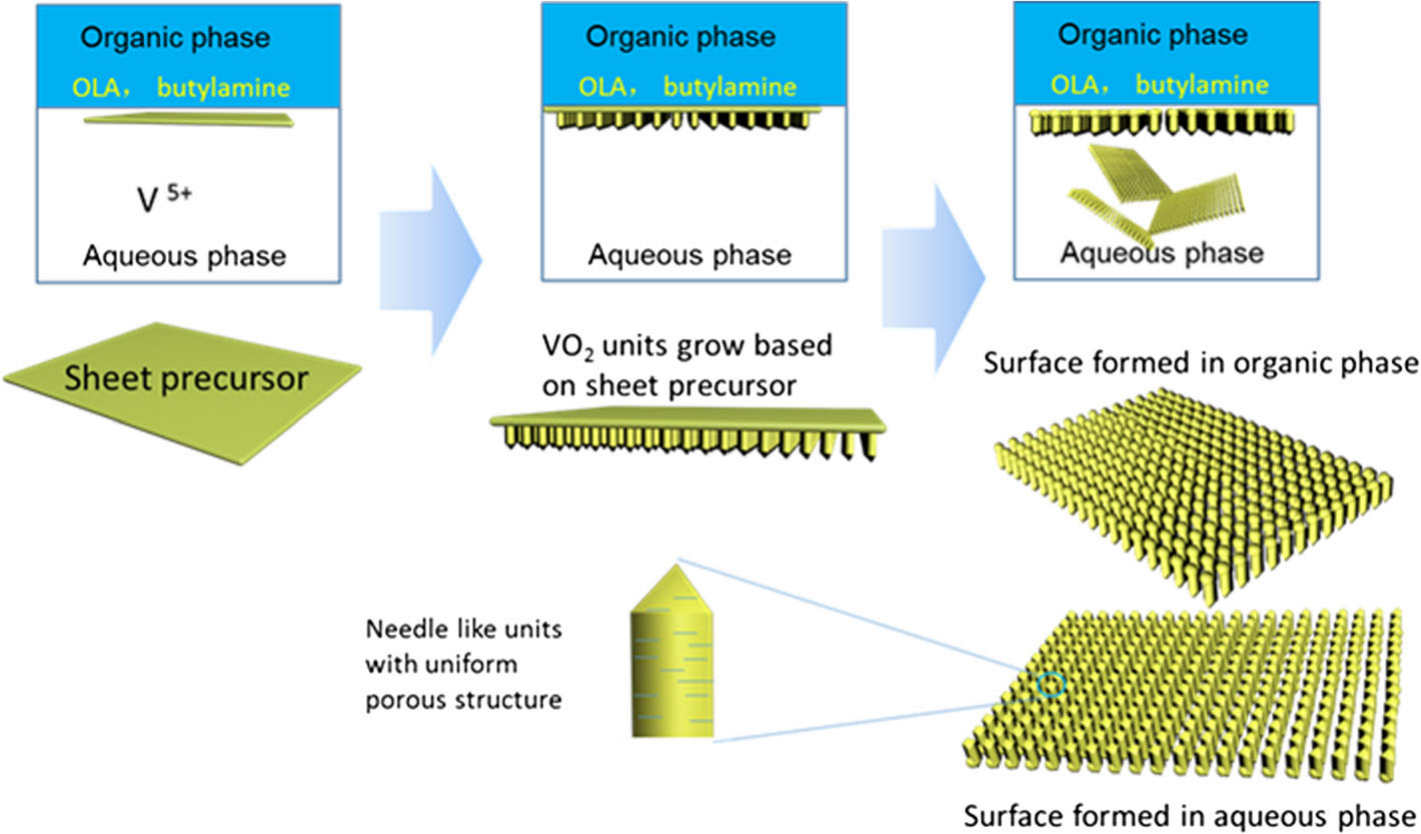
Illustration of the formation process of 2D VO_2_ mesoporous microarrays

Preparation process of 2D VO_2_ microarrays was illustrated in Scheme 1. V_2_O_5_ was first dissolved in a H_2_O_2_ aqueous solution and used as the aqueous phase. Toluene solution contained oleic acid, and *tert*-butylamine was used as the organic phase. The aqueous and organic solution will not dissolve each other and form an aqueous–organic liquid interface. This liquid interface was used as template for the formation of 2D VO_2_ microarrays. Under hydrothermal conditions, *tert*-butylamine dissolved into aqueous solution to enhance the pH value, and thus, V^5+^ will be reduced by oleic acid at the liquid interface. As shown in Scheme 1, VO_2_ nanosheets were first formed at the liquid interface, and then, needle-like VO_2_ units with a mesoporous structure were grown on the nanosheets in aqueous phase at the liquid interface. Through the growth of needle-like VO_2_ units, the nanosheets formed transformed into the aggregates of nanoparticles in organic phase, and therefore, 2D microarrays were finally formed.

**Fig. 1:**
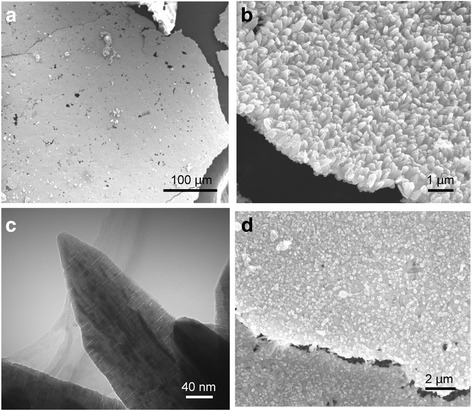
SEM images of the VO_2_ 2D microarrays (**a**) and the surfaces formed in aqueous (**b**) and organic (**d**) phase. TEM image of the mesoporous VO_2_ units (**c**)

**Fig. 2:**
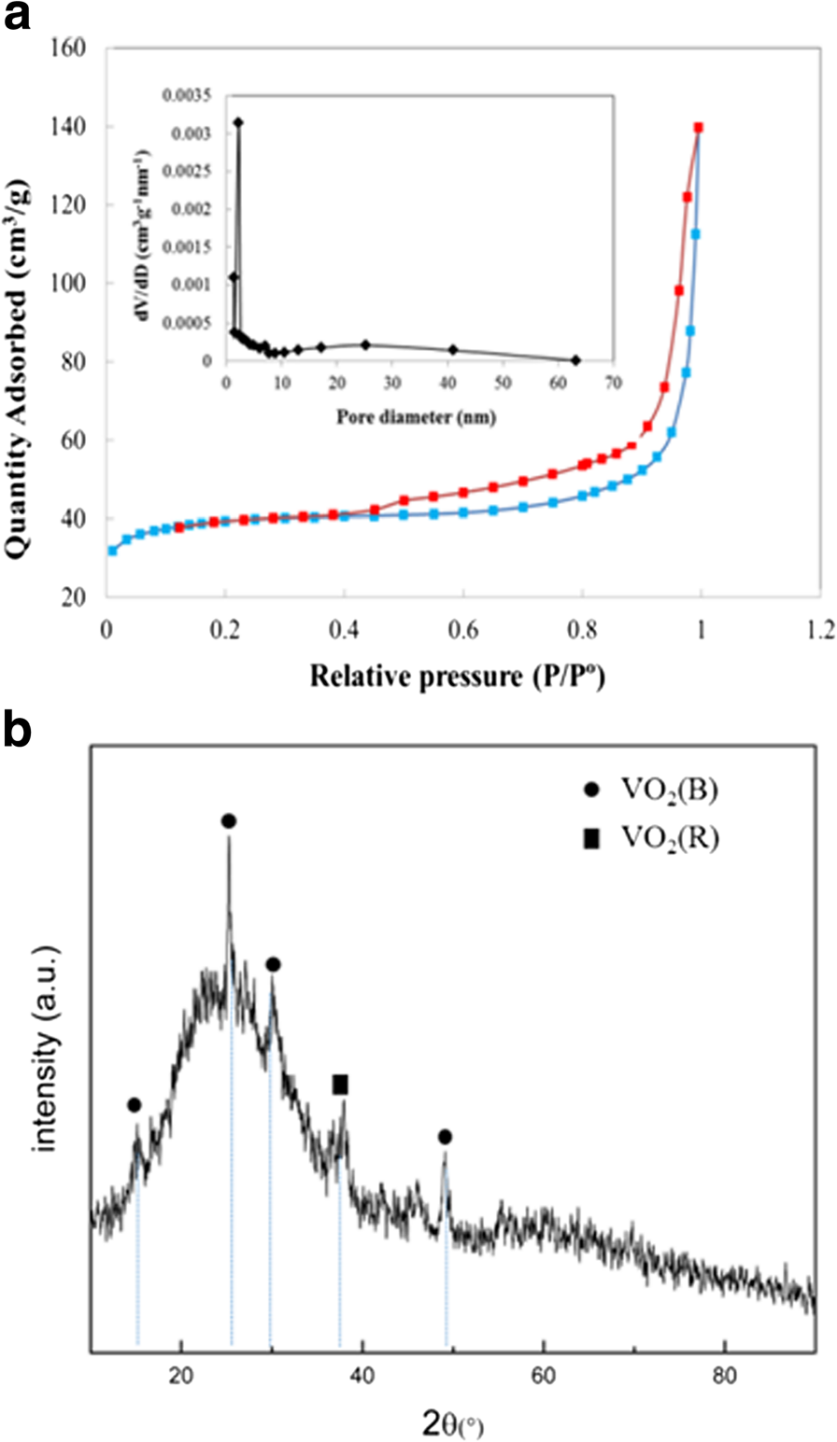
N_2_ adsorption–desorption isotherms with corresponding pore size distribution (**a**) and XRD pattern of 2D VO_2_ microarrays (**b**)

Figure 1a displayed the SEM image of the 2D VO_2_ microarrays (designated as VO_2_-N microarrays), in which the microarrays exhibited uniform structure with a size over several millimeters. At high magnification (Fig. 1b,d and Additional file 1: Figure S1), two different surfaces formed in aqueous and organic phase at the liquid interface. Figure 1b shows the surface formed in aqueous phase. It can be seen that the 2D microarrays were composed of edge-shared needle-like units. The thickness of the microarrays was about 1 μm. As to the needle-like VO_2_ unit, the width of ca. 350 nm and the length of 1 μm were obtained (Fig. 1c and Additional file 1: Figure S1c, d). Figure 1c showed the TEM image of the VO_2_ needle-like units. The size of the particles was about 1 μm, which agrees with the SEM observation. The electron diffraction (ED) pattern of the particle indicated a single crystal nature. It can be identified that the needle-like units have a uniform porous structure. Pores with a uniform size of 2 nm were distributed on the needle-like particles. The depth of the pores ranged from 20 to 100 nm, and the width was about 20 nm. The Brunauer–Emmett–Teller (BET) specific surface area and porosity explored by nitrogen adsorption-desorption analysis were shown in Fig. 2a. Deducing from the nitrogen adsorption/desorption isotherm curve, the surface area of the 2D microarray was 80 m^2^/g, attributed to the type IV isotherm with a H1 hysteresis loop [43, 44]. As shown in Fig. 2a, the 2D microarray displayed a narrow pore size distribution, which mainly ranged from 1.9 to 3.8 nm with an average pore diameter of 2.85 nm. The corresponding pores should be mainly related to those located in the needle-like units, as revealed in Fig. 1c. These results suggested that the 2D microarrays were a typical mesoporous structure, which could provide uniform short and fast ion diffusion pathway for high performance in supercapacitors. Figure 1d and Additional file 1: Figure S1a, b showed the surface of 2D VO_2_ microarrays formed in organic phase at the liquid interface. This surface was composed by irregular particles with a size of ca. 200 nm. Figure 2b showed the XRD pattern of the microarrays. The diffraction peaks at 16°, 25°, 30°, and 49° corresponded to the (200), (110), (− 401), and (312) crystal faces of VO_2_ (B) phase (JCPDS no. 31-1438) [45], resepectively, while the diffraction peaks at 37° corresponded to the (011) crystal face of VO_2_ (R) phase. This result indicated that the VO_2_ microarrays were a mixture of VO_2_ (B) and VO_2_ (R) phases, and the main phase was VO_2_ (B), which is desirable for high-performance capacitances.

The 2D VO_2_ microarrays showed a unique multi-structure formed in aqueous and organic phases in this work. This unique structure can be attributed to the inorganic–organic liquid interface. Additional file 1: Figure S2 shows the kinetics of the formation of 2D microarrays. When synthesized for 1 h, millimeter-sized sheets with a thickness of ca. 100 nm were obtained (Additional file 1: Figure S2a). In TEM (Additional file 1: Figure S2b, c), the sheet has a single crystal nature and considerable nanocrystals with a size of 5 nm were observed on its surface. In aqueous phase, the nanocrystals formed on the sheet surface were the seeds for promoting the growth of needle-like VO_2_ units. Additional file 1: Figure S2d, e displays the SEM images synthesized for 8 h. Particles with irregular morphology growing on the sheets were observed in aqueous phase. When synthesized for 16 h, some of the particles possessed similar morphology to that of the VO_2_ needle-like units (Additional file 1: Figure S2f). These observations suggested that the VO_2_ needle-like units grew on the firstly formed sheet in aqueous solution, and then, the sheets transformed into the aggregates of irregular particles in organic phase (Fig. 1c and Additional file 1: Figure S1).

The morphology of the 2D microarrays can be controlled by changing the solvent, reducer, and surfactant. Additional file 1: Figure S3 shows the VO_2_ microarrays synthesized using ultrapure water as the aqueous phase (designated as VO_2_-S). The low dielectric constant of ultrapure water will delay the nucleation and growth of VO_2_ particles. After the synthesis, the sheet formed in organic phase did not disappear, and flowers composed of nanosheet were observed from the surface formed in aqueous solution. The nanosheets have a size over 30 μm and a thickness of 100 nm, and needle-like particles were not observed. Additional file 1: Figure S4 showed the VO_2_ microarrays (designated as VO_2_-F microarrays) using hydrazine added in aqueous solution as the reducer. 2D microarrays were also obtained for the samples synthesized using hydrazine as the reducer, and on the other hand, the VO_2_ units changed into a fusiformis-like morphology. The fusiformis-like units self-assembled into rod-like aggregates as shown in Additional file 1: Figure S4b, c. It is worthy noting that no porous structure was identified for the fusiformis-like and nanosheet units synthesized using hydrazine and ultrapure water as shown in Additional file 1: Figures S3e and S4d. When oleylamine was used instead of butylamine, nanocubes with a size of 200 nm dispersed in toluene solution were obtained and no microarrays were observed at the liquid interface as shown in Additional file 1: Figure S5.

**Fig. 3:**
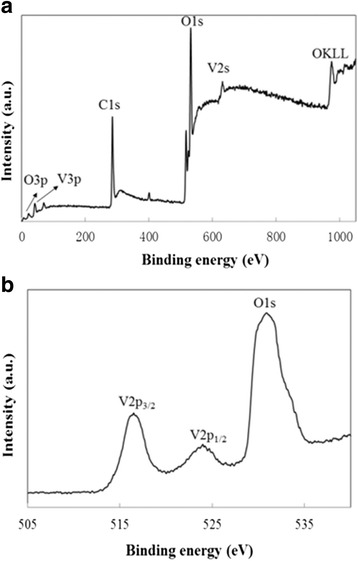
XPS spectra: survey scan. **a** V 2p and **b** O of 2D VO_2_ microarrays

Figure 3 shows the XPS spectra of 2D VO_2_-N microarrays. In the survey region, carbon, vanadium, and oxygen were detected (Fig. 3a). The ratio of the O atom and the V atom was about 2, which is in good agreement with the stoichiometric ratio of VO_2_. Figure 3b shows the core level binding energy for V (2p) peaks. The binding energies for V 2p_3/2_ and 2p_1/2_ observed at 516.7 and 524.6 eV agreed well with those of V^4+^ ion, and no other peaks belong to V^5+^ were detected [46].

**Fig. 4:**
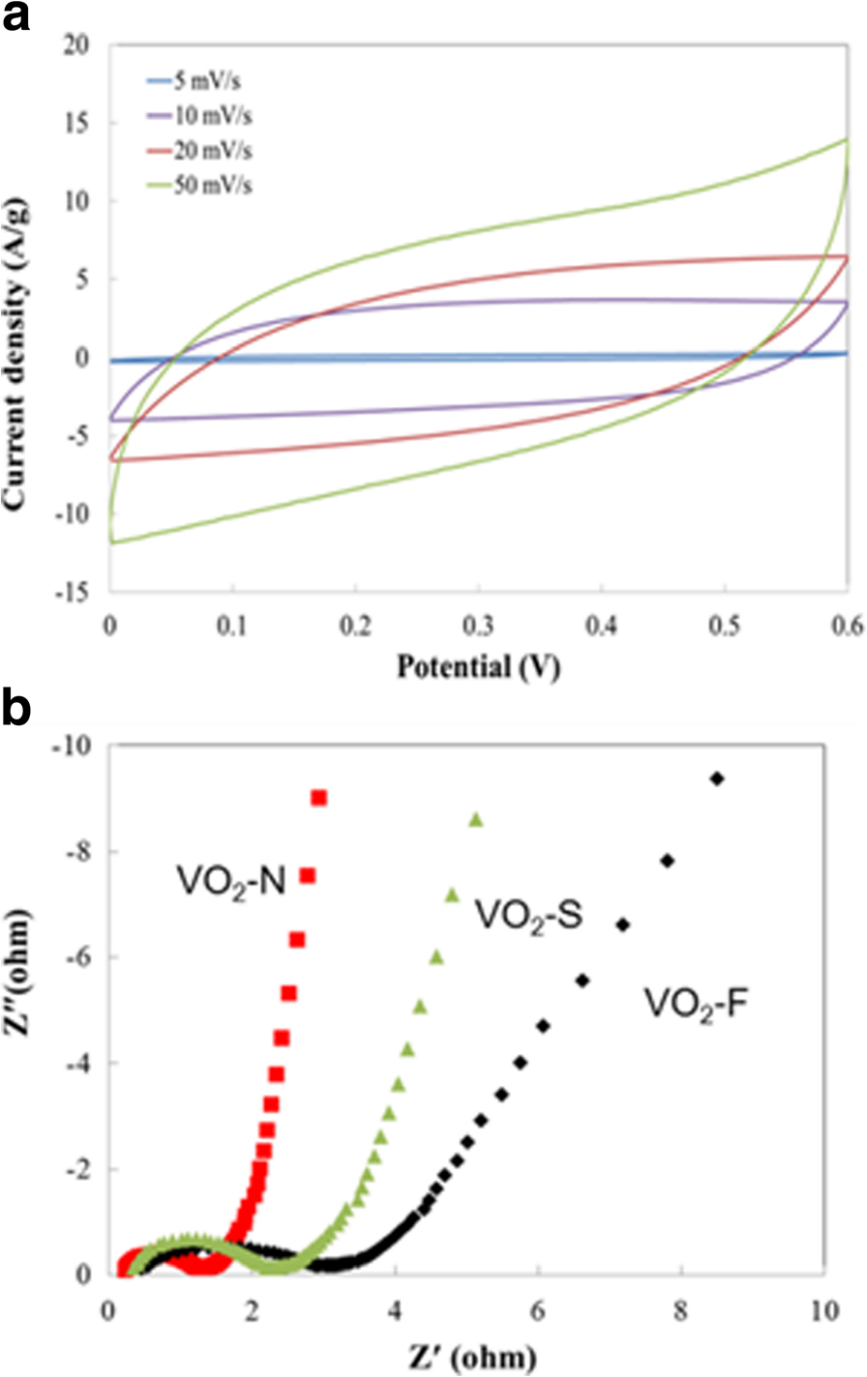
CV curves at the scan rates of 5–50 mV/s (**a**) and EIS spectra of 2D VO_2_ microarrays (**b**)

Cyclic voltammograms (CV) were measured to characterize the supercapacitor performance of the VO_2_-N microarrays (Fig. 4a). The CV curves retained a similar rectangular shape even at high sweep rate. The symmetrical shapes observed in CV curves at different scan rates indicated that the redox reaction is highly reversible and responsible for the enhanced capacitance performance. Electrochemical impedance spectrum (EIS) test was used to investigate the kinetics of charge carrier transport (Fig. 4b). The straight line at low frequency deduced from the Warburg impedance. The VO_2_-N microarrays displayed a sharply increased slope closing to 90°, implying the ideal capacitive behavior and short diffusion resistance of electrolyte ions in the electrode. At high-frequency region, the semicircle came from the resistance in parallel with capacitance. The semicircle was identified for all the three types of 2D microarrays, which originated from the charge transfer process of Faradaic reactions. The VO_2_-N microarrays exhibited the lowest diminished equivalent series resistance (ESR) of 1.07 Ω. The considerably depressed semicircle and low inside resistance suggested rapid ion transport within the VO_2_-N microarray electrode.

**Fig. 5:**
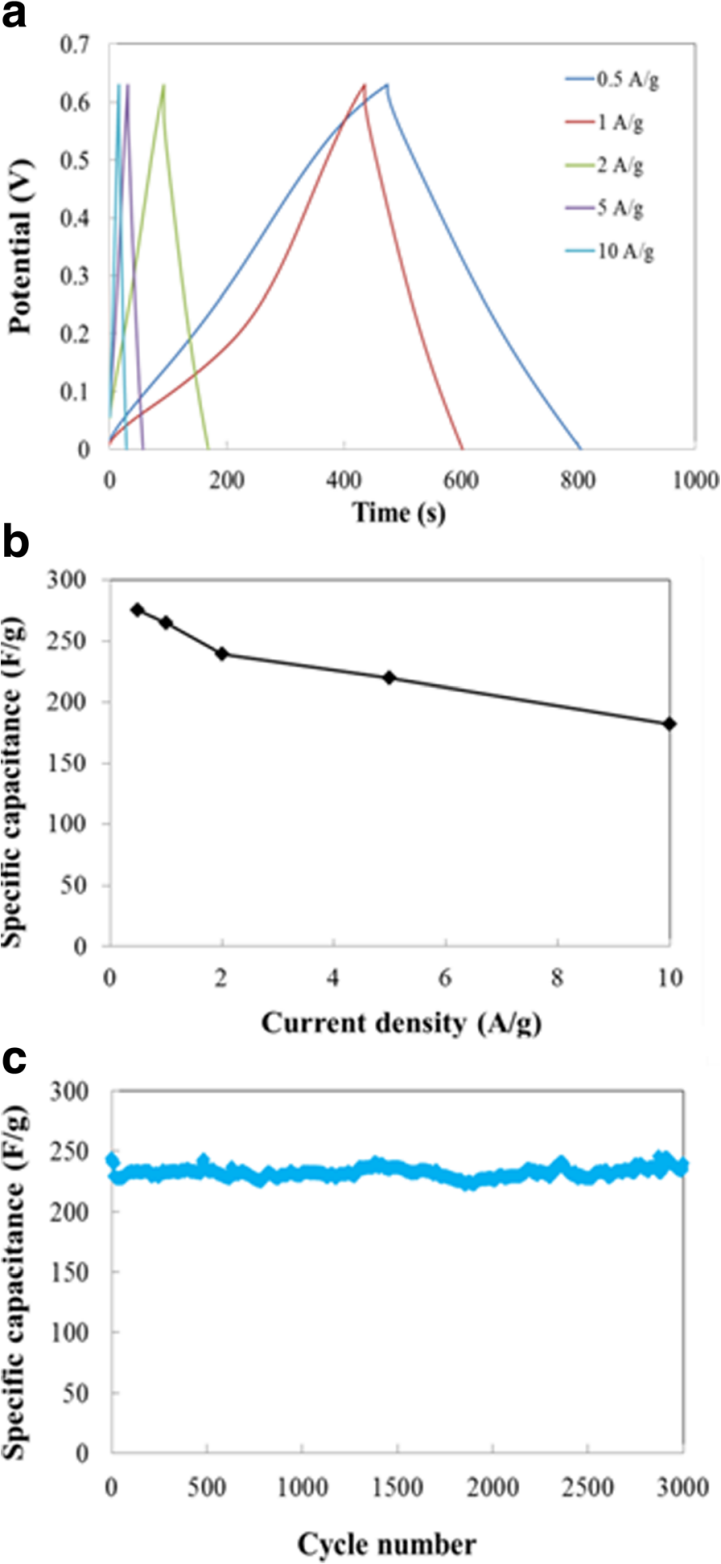
**a** Charge–discharge curves at the current density between 0.5 and 10 A/g, **b** corresponding specific capacitance, and **c** cycling performance of 2D VO_2_ microarrays at 1 A/g

Figure 5a showed the galvanostatic charge-discharge curves of the VO_2_-N microarray electrode at the current density ranged from 0.5 to 10 A/g, and the corresponding specific capacitances were illustrated in Fig. 5b. Within the whole current density range, the VO_2_-N microarray electrode yielded high specific capacitances. The capacitance of 275 F/g was obtained at 0.5 A/g, and the capacitance of 265 F/g at 1 A/g obtained a capacitance retention of 96% for comparison to that at 0.5 A/g. At 10 A/g, the capacitance was 182 F/g which maintained a capacitance retention of 66%. The long-term cycling behavior of the capacitive performance was examined up to 3000 cycles at a current density of 2 A/g (Fig. 5c). No capacitance fading was observed during cycling for VO_2_-N microarray electrode, and the capacitance of 239 F/g maintained unchanged after 3000 cycles. Meanwhile, in other types of microarrays without the mesoporous structure, the specific capacitances were only 96 and 64 F/g (1 A/g) for the VO_2_-S and VO_2_-F 2D microarrays, respectively (Additional file 1: Figure S6c). The capacitance thus decreased rapidly to 73 F/g only after 300 cycles at 1 A/g for VO_2_-S microarray as shown in Additional file 1: Figure S6.

It can be identified that the VO_2_-N microarray obtained excellent capacitance performances. Up to now, the highest capacitance of pure VO_2_ was 180 F/g at a current density of 1 A/g [35]. The capacitance of VO_2_-N microarray reached to 265 F/g at 1 A/g, and the capacitance retention was high at high current density (182 F/g at 10 A/g). Furthermore, the cycle performance of the microarray was excellent. In general, the cycle performance of pure VO_2_ was very poor due to its low electrical conductivity; the capacitance retention decreased to about 60% after 500 cycles [28–35]. On the other hand, No capacitance fading was observed during cycling for VO_2_-N microarray electrode after 3000 cycles at a high current density (2 A/g). For the Faradaic effect-based pseudocapacitance, ion intercalation and reaction were the dominant phenomenon near the surface, little contribution from the inside of the particle to capacitance. Large specific surface area will significantly maximize the specific capacitance, with the further contribution from the double layer capacitance effect. In this work, the uniform mesoporous structure of the VO_2_ units in the VO_2_-N 2D microarrays provided high surface area and short ion diffusion pathway for realizing large specific capacitance. In other types of microarrays, however, we did not observe a mesoporous structure (Additional file 1: Figures S3 and S4), and their BET surface areas were only 21 and 13 m^2^/g for VO_2_-S and VO_2_-F 2D microarrays, respectively. Furthermore, the VO_2_-N 2D microarray obtained the higher conductivity compared to the VO_2_-S and VO_2_-F 2D microarrays, yielding excellent cycle performance of VO_2_-N 2D microarray.

## Conclusions

In summary, we report a facile way to fabricate the 2D VO_2_ microarrays. The organic-inorganic liquid interface acted as a soft template for the formation of the microarrays. The morphology of the units can be controlled by changing the solvent and reducer. Needle-like nanosheets and fusiformis-like units were obtained. As the supercapacitor electrode, the 2D VO_2_ microarrays of needle-like units exhibited high specific capacitance, remarkable rate capability, and excellent cycle performance. The mesoporous structure of the needle-like units and high conductivity of the microarrays contributed to the excellent capacitance performance.

## Additional file

Additional file 1: Supplementary data associated with this article can be found in the online version. (DOCX 1589 kb)

## Abbreviations

2D: Two-dimensional
AB: Acetylene black
BET: Brunauer–Emmett–Teller
CNT: Carbon nanotube
CV: Cyclic voltammetry
ED: Electron diffraction
EDL: Electrical double-layer capacitors
EIS: Electrochemical impedance spectrum
ESR: Equivalent series resistance
NMP: *N*-methyl-2-pyrrolidone
PVDF: Polyvinylidenedifluoride
rGO: Reduced graphene oxide
TMOs: Transition metal oxides
XRD: X-ray diffraction
