# Supplementary material for: Two-Dimensional VO2 Mesoporous Microarrays for High-Performance Supercapacitor
Source: Nanoscale Res Lett. 2018 May 8;13:142. doi: 10.1186/s11671-018-2557-7 (PMC5940969; doi:10.1186/s11671-018-2557-7)
Supplement: Supplementary file 1 — Supplementary data associated with this article can be found in the online version. (DOCX 1589 kb) [file 11671_2018_2557_MOESM1_ESM.docx]

**Supporting Information**

**Two-dimensional mesoporous VO_2_ microarrays for high performance supercapacitor**

Yuqi Fan, ^1,2*^ Delong Ouyang,^1,2^ Bao-Wen Li,^3*^ Feng Dang, ^4^ Zongming Ren^1,2^

^1^ College of Geography and Environment, Shandong Normal University, Jinan 250014, China.

^2^ Institute of Environment and Ecology, Shandong Normal University, Jinan 250014, China.

^3^ Schools of Materials Science and Engineering, Wuhan University of Technology, Wuhan 430070, China.

^4^ Key Laboratory for Liquid-Solid Structural Evolution and Processing of Materials, Shandong University, Jinan 250061, China.

Corresponding authors: bwli@whut.edu.cn; yuqifan@sdnu.edu.cn


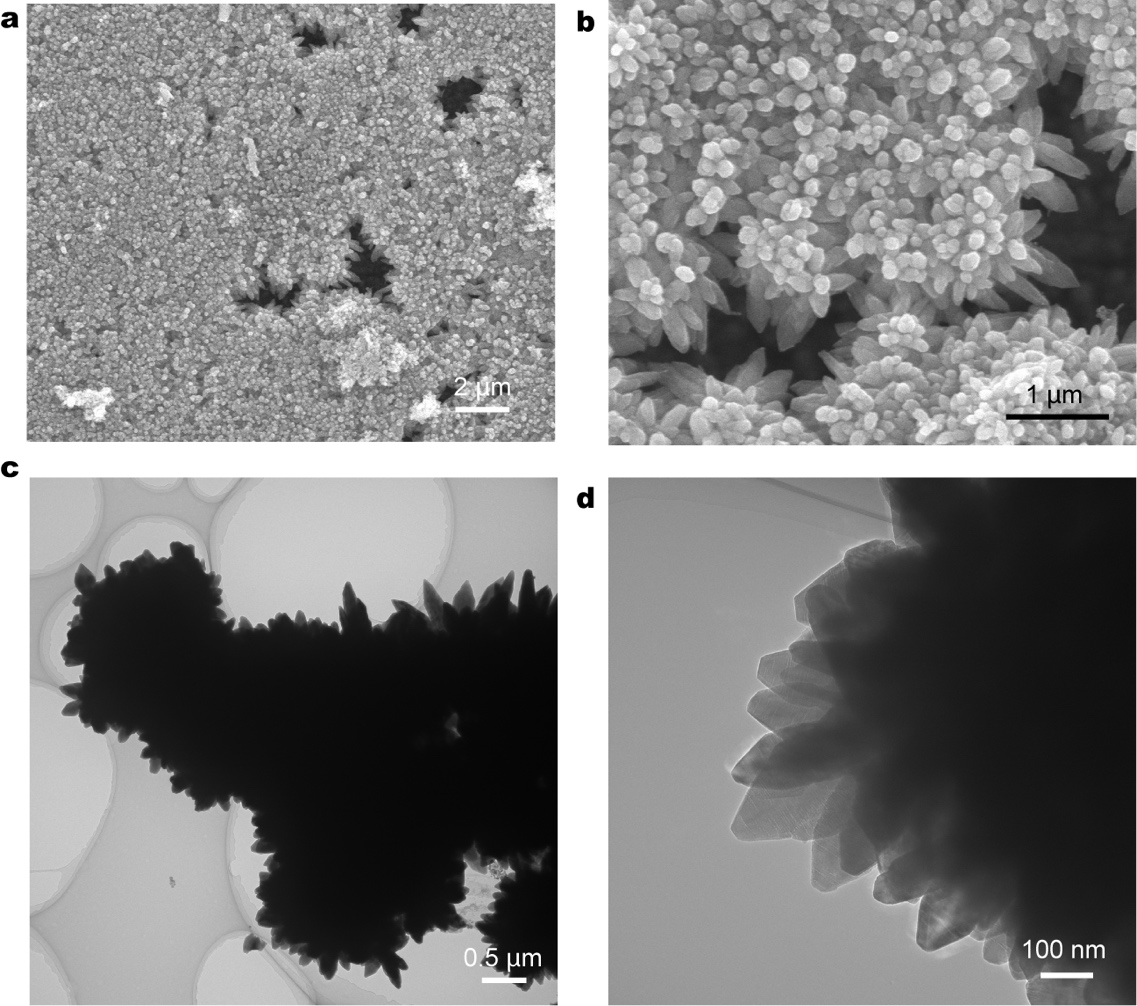


Figure S1. SEM (a, b) and TEM images (c, d) of VO_2_-N 2D microarrays.


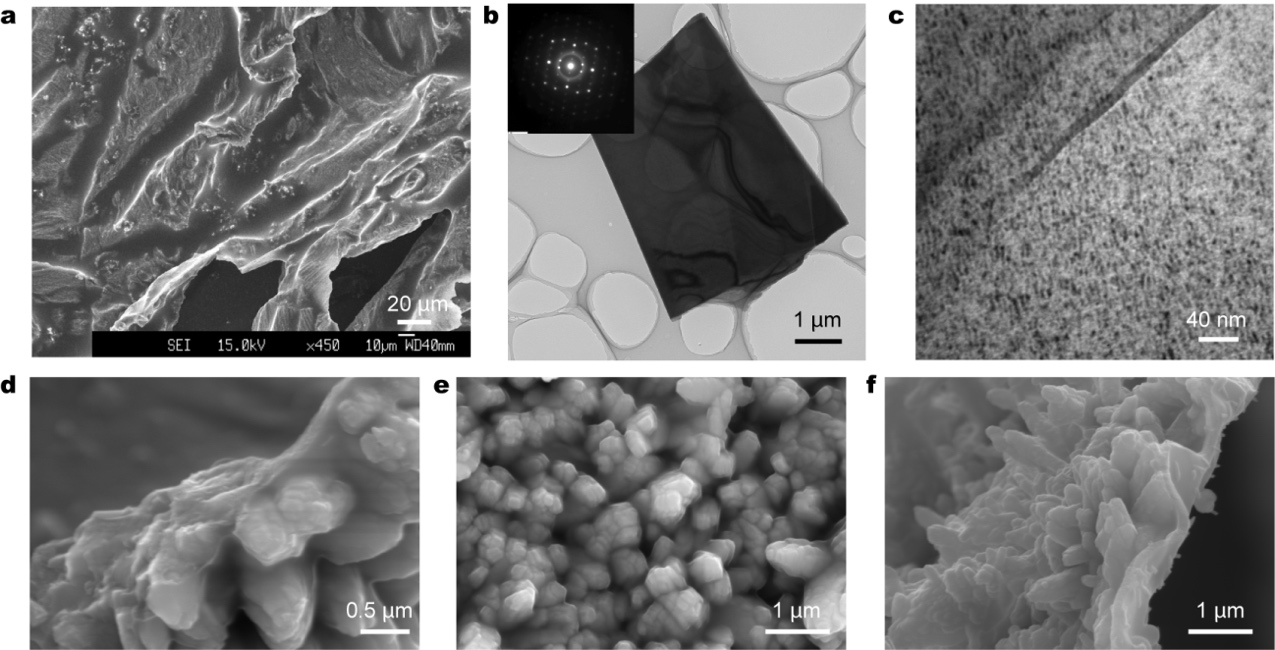


Figure S2. SEM and TEM images of VO_2_-N 2D microarrays synthesized for 1 (a-c), 8 (d, e) and16 h (f).


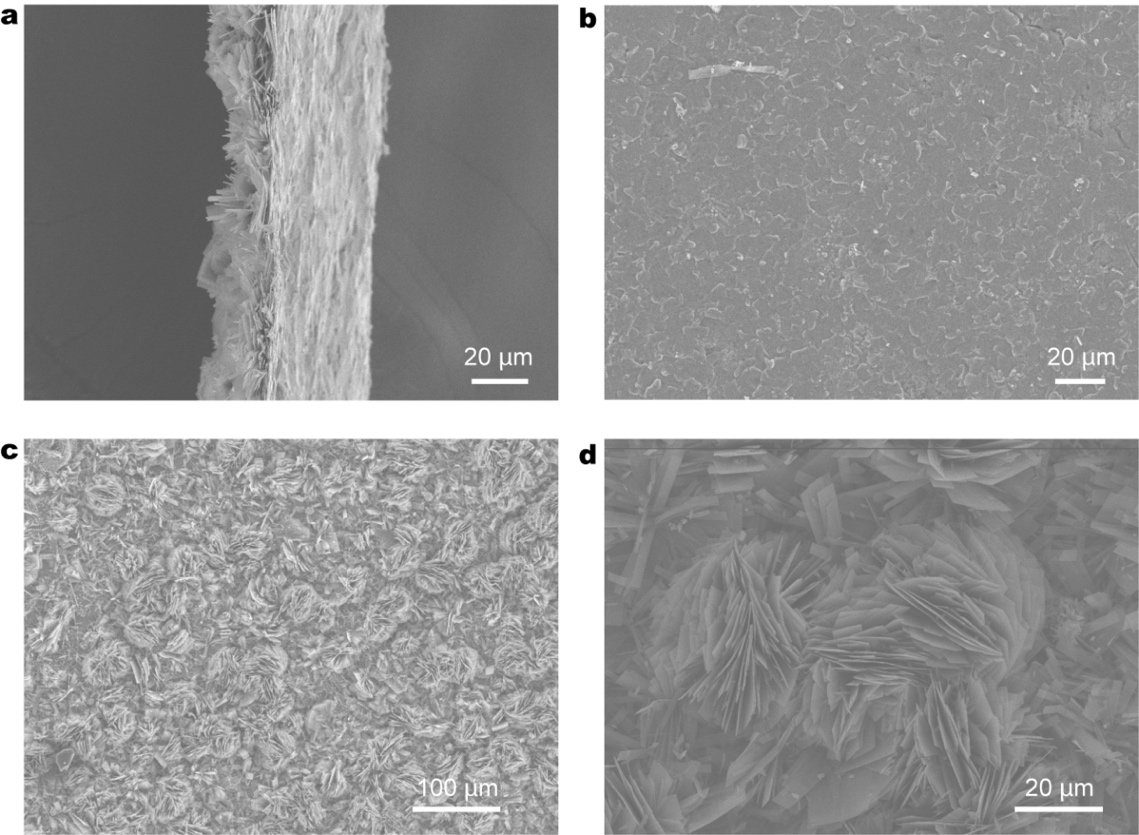


Figure S3. SEM images of 2D VO_2_-S microarrays synthesized using ultrapure water as the aqueous phase.


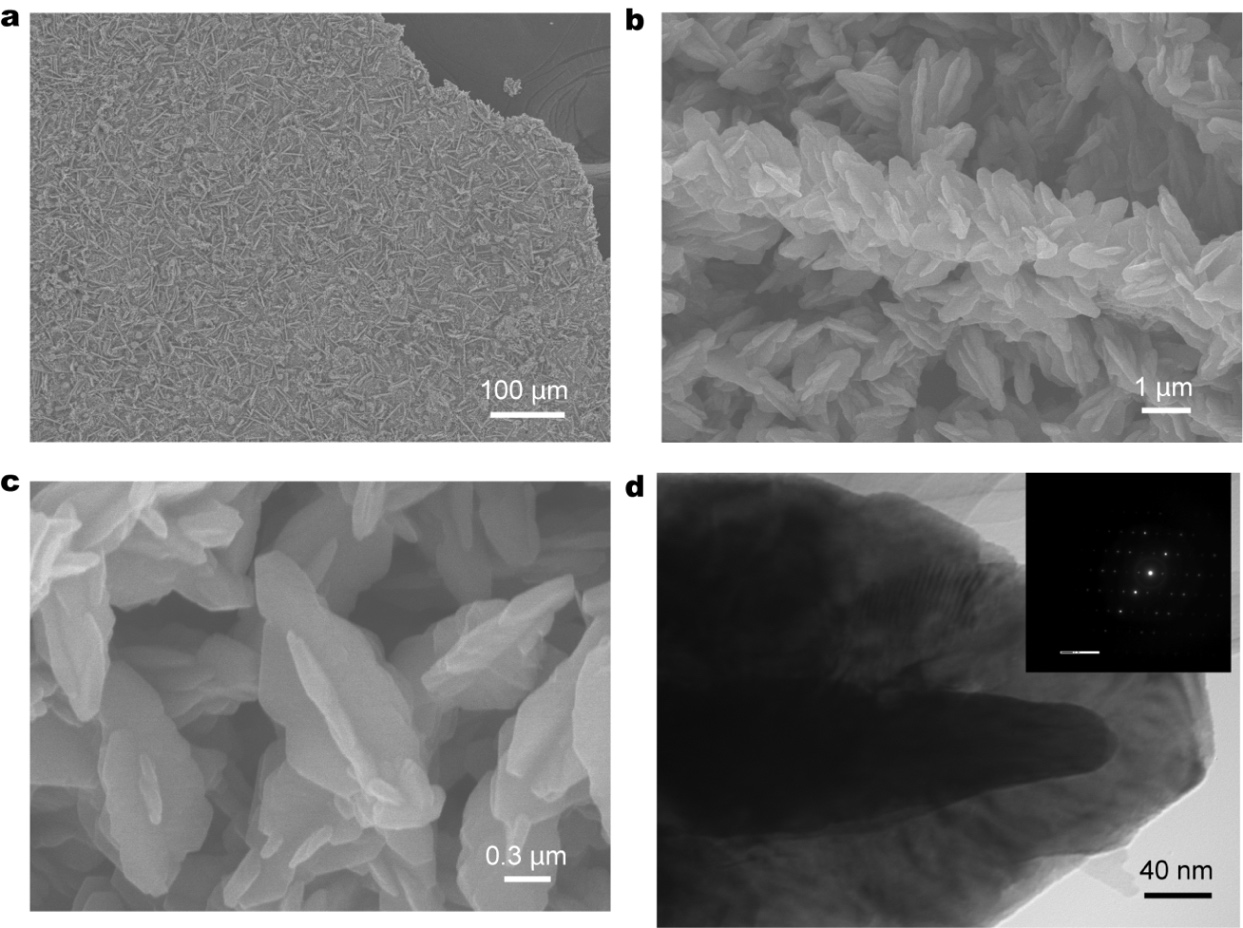


Figure S4. SEM and TEM images of 2D VO_2_-F microarrays synthesized using hydrazine as the reducer in aqueous phase.


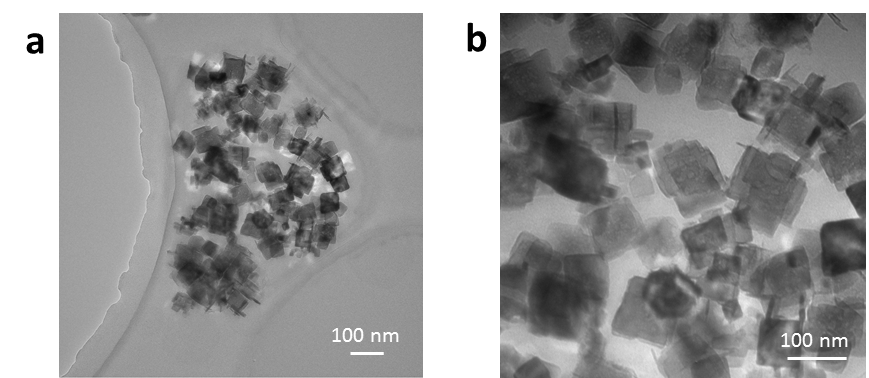


Figure S5. TEM images of the VO_2_ nanocubes using oleyamine as the surfactant.


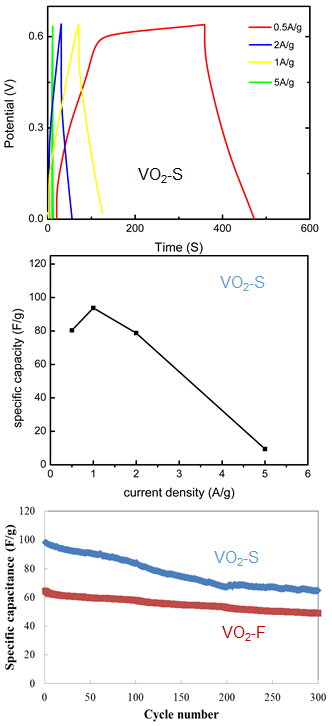


Figure S6. (a) Charge-discharge curves at the current density ranging from  0.5 to 5 A/g, (b) corresponding specific capacitance  of 2D VO_2_-S microarrays and (c) cycling performance of 2D VO_2_-S and VO_2_-F microarrays at a current density of 1.0 A/g.
